# Systematic review and narrative synthesis of surgeons' perception of postoperative outcomes and risk

**DOI:** 10.1002/bjs5.50233

**Published:** 2019-11-26

**Authors:** N. M. Dilaver, B. L. Gwilym, R. Preece, C. P. Twine, D. C. Bosanquet

**Affiliations:** ^1^ Aneurin Bevan University Health Board, Royal Gwent Hospital Newport UK; ^2^ Academic Section of Vascular Surgery, Department of Surgery and Cancer Imperial College London London UK; ^3^ Division of Population Medicine Cardiff University Cardiff UK; ^4^ Southmead Hospital, North Bristol NHS Trust Bristol UK

## Abstract

**Background:**

The accuracy with which surgeons can predict outcomes following surgery has not been explored in a systematic way. The aim of this review was to determine how accurately a surgeon's ‘gut feeling’ or perception of risk correlates with patient outcomes and available risk scoring systems.

**Methods:**

A systematic review was undertaken in accordance with PRISMA guidelines. A narrative synthesis was performed in accordance with the Guidance on the Conduct of Narrative Synthesis In Systematic Reviews. Studies comparing surgeons' preoperative or postoperative assessment of patient outcomes were included. Studies that made comparisons with risk scoring tools were also included. Outcomes evaluated were postoperative mortality, general and operation‐specific morbidity and long‐term outcomes.

**Results:**

Twenty‐seven studies comprising 20 898 patients undergoing general, gastrointestinal, cardiothoracic, orthopaedic, vascular, urology, endocrine and neurosurgical operations were included. Surgeons consistently overpredicted mortality rates and were outperformed by existing risk scoring tools in six of seven studies comparing area under receiver operating characteristic (ROC) curves (AUC). Surgeons' prediction of general morbidity was good, and was equivalent to, or better than, pre‐existing risk prediction models. Long‐term outcomes were poorly predicted by surgeons, with AUC values ranging from 0·51 to 0·75. Four of five studies found postoperative risk estimates to be more accurate than those made before surgery.

**Conclusion:**

Surgeons consistently overestimate mortality risk and are outperformed by pre‐existing tools; prediction of longer‐term outcomes is also poor. Surgeons should consider the use of risk prediction tools when available to inform clinical decision‐making.

## Graphical abstract

The aim of this review was to determine how accurately surgeons' perceptions of risk correlate with patient outcomes and available risk scoring systems. An extensive systematic review and narrative synthesis was undertaken. Twenty‐seven studies comprising 20 898 patients were included. Surgeons were outperformed by pre‐existing tools.

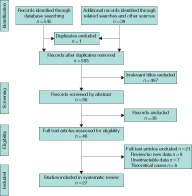

Gut feeling outperformed by prediction tools

## Abstract (es)

**Antecedentes:**

La precisión con la cual los cirujanos pueden predecir los resultados de la cirugía no se ha estudiado de forma sistemática. El objetivo de esta revisión fue determinar con qué precisión la intuición de un cirujano o su percepción del riesgo se correlacionaba con los resultados del paciente y con los sistemas de puntuación del riesgo disponibles.

**Métodos:**

Se efectuó una revisión sistemática siguiendo las directrices PRISMA. Se realizó una síntesis narrativa de acuerdo con la guía para la realización de síntesis narrativas en revisiones sistemáticas. Se incluyeron los estudios que comparaban las evaluaciones preoperatorias o postoperatorias de los cirujanos respecto a los resultados de los pacientes. También se incluyeron aquellos estudios en los que se hacían comparaciones con herramientas de puntuación de riesgo. Se evaluaron la mortalidad postoperatoria, la morbilidad global y la morbilidad específica de las intervenciones, y los resultados a largo plazo.

**Resultados:**

Se incluyeron 27 estudios con 20.898 pacientes en los que se realizaron procedimientos de cirugía general, digestiva, cardiotorácica, ortopédica, vascular, urológica, endocrina y neurocirugía. Los cirujanos predijeron consistentemente mayores tasas de mortalidad, siendo superados en precisión por los sistemas de estimación del riesgo existentes en seis de los siete estudios que utilizaron el área bajo la curva (*area under curve*, AUC) operativa del receptor. La predicción de la morbilidad general por parte de los cirujanos fue buena y era equivalente, incluso mejor, que los modelos de predicción de riesgos preexistentes. La capacidad de los cirujanos para predecir los resultados a largo plazo fue pobre, con una AUC que oscilaba entre 0,51 y 0,75. Cuatro de cinco estudios encontraron que las estimaciones de riesgo postoperatorias fueron más precisas que las realizadas preoperatoriamente.

**Conclusión:**

Los cirujanos sobrestiman consistentemente el riesgo de mortalidad, siendo superados en precisión por las herramientas preexistentes. La predicción de resultados a largo plazo también es muy pobre. Los cirujanos deberían considerar el uso de herramientas de predicción de riesgo cuando estén disponibles para informar en el proceso de decisión clínica.

## Introduction

Surgical procedures all carry associated risks. It is therefore important that surgeons are able to make accurate predictions of potential benefit and risk, including immediate mortality and morbidity, as well as long‐term outcomes, to enable balanced decision‐making and fully informed consent. Risks can also be estimated after surgery, based on additional perioperative and intraoperative data, which allows contemporary prediction of outcome. There are numerous risk prediction models that enable the surgeon to quantify risk based on measurable parameters^1^, ^2^, ^3^, ^4^, ^5^. However, there are inherent limitations in using a generalized risk prediction model, which may not include clinical data pertinent to the individual case in question, leading to variability in model accuracy^6^, ^7^, ^8^, ^9^, ^10^.

As a result, risk prediction tools are generally used in tandem with the surgeon's ‘gut feeling’ of overall risk and anticipated outcome (‘clinical gestalt’). Several disparate factors influence surgeons' perception of outcome: patient factors, such as their perceived fitness, their pathology and planned procedure; setting factors, such as the experience of other members of staff; and surgeon factors, such as clinical knowledge, operative skill, previous significant surgical complications, and inclinations and attitudes^11^, ^12^, ^13^.

Anticipating surgical risk is subject to multiple biases, which make it challenging. These include the natural tendency toward anecdotal recall and the availability heuristic (the likelihood of making a decision based on how easily the topic or examples come to mind)^14^, ^15^. Some studies^16^, ^17^, ^18^ support the accuracy and reproducibility of surgeons' predictions, whereas others^19^, ^20^, ^21^, ^22^ demonstrate less favourable results. The complexity of synthesizing risk perceptions is significant and incompletely understood^23^, ^24^. The accuracy of surgeons' prediction has not been explored previously in a systematic manner.

The aim of this review was thus to determine, from the available evidence, whether a surgeon's gut feeling or perception of risk correlates with postoperative outcomes, and to compare this prediction with currently available risk scoring systems, where available.

## Methods

This systematic review was undertaken in accordance with the PRISMA guidelines^25^, ^26^. MEDLINE (via PubMed), Embase, the Cochrane Library Database, and the Cochrane Collaboration Central Register of Controlled Clinical Trials were searched with no date or language restrictions, with the last search date on 9 July 2018. The search term used was (‘Surgeons’[Mesh] OR ‘General Surgery/manpower*’ [MeSH]) AND (‘perception’ OR ‘intuition’ OR ‘predict*’ OR ‘decision making’ [mesh]). There was no restriction on publication type. This search was complemented by an exhaustive review of the bibliography of key articles, and also by using the Related Articles function in PubMed of included papers. Results were restricted to human research published in English.

### Inclusion and exclusion criteria

All studies of patients undergoing surgery in which a preoperative or postoperative surgeon assessment (or proxy assessment) of a postoperative outcome was performed were included. This included articles that reported general risk (such as mortality) or a surgery‐specific risk (for example anastomotic leakage). Studies that made comparisons with established risk scoring tools were also included. Papers or abstracts in English, or non‐English papers with an English abstract, were included.

Papers describing the risk assessment of ‘theoretical’ cases, or patient vignettes in a situation distant from clinical practice (such as a conference), were excluded, as were studies in which surgeons' assessment of risk was compared with an established risk scoring tool, without data on actual patient outcome.

### Data extraction and assessment of study quality

Three authors independently extracted data and assessed the methodological quality of the studies, with all data extraction independently checked by the senior author.

The following baseline data were extracted from each study: first author, year of publication, data collection period, geographical location, study design and type (single or multiple centres, number of surgeons involved in risk estimation, whether consecutive patients were enrolled), surgical specialty, whether other risk scoring systems were used for comparison and, if so, whether the assessor was blinded to this result. Data extracted regarding the assessment of risk included: risk outcome assessed; timing of risk estimation (preoperative or postoperative); type of risk assessment by surgeons (qualitative, quantitative, continuous scale such as a visual analogue scale (VAS), or composite score); absolute value of risk event predicted by surgeon and by scoring system; absolute value of risk occurrence rate; summary data on outcome reported, including area under the curve (AUC) of receiver operating characteristic (ROC) curves, observed : expected (O : E) or predicted : observed (P : O) ratios, or any other summary data.

When data were available, AUCs were extracted with their 95 per cent confidence intervals. AUCs greater than 0·9 were considered as indicating high performance, 0·7–0·9 as moderate performance, 0·5–0·7 as low performance, and less than 0·5 as indicating risk assessment no better than chance alone^27^, ^28^.

Risk predictions made by pre‐existing tools, such as the Physiological and Operative Severity Score for the enumeration of Mortality and morbidity (POSSUM)^1^, Portsmouth‐POSSUM (P‐POSSUM)^4^ or Continuous Improvement in Cardiac Surgery Program (CICSP)^5^, were compared with outcome when given. Internal prediction models, where authors would derive significant predictive co‐variables from their data set and assess the accuracy of these co‐variables within the same data set, were not evaluated as they lacked validity.

Study quality was assessed using the Newcastle–Ottawa (NO) score^29^, ^30^. The NO score assigns points based on: the quality of patient selection (maximum 4 points); comparability of the cohort (maximum 2 points); and outcome assessment (maximum 3 points). Studies that scored 6 points or more were considered to be of higher quality.

### Outcome measures

The following outcome measures were defined *a priori* and refined during data extraction: postoperative mortality (usually defined as 30 days after surgery); postoperative general morbidity (usually defined as 30 days after surgery); postoperative procedure‐specific morbidity; and long‐term outcome (typically operation‐specific).

Further comparative analyses of outcomes included comparison of preoperative and postoperative predictions, and of predictions made by consultants and surgical trainees.

### Narrative synthesis

Given the marked heterogeneity in study design, patient population included, method of assessing risk and outcomes assessed, meta‐analysis was deemed not appropriate. A narrative synthesis was therefore performed according to the Guidance on the Conduct of Narrative Synthesis In Systematic Reviews^31^. Three authors systematically summarized each article using bullet points to document key aspects of each study, focusing particularly on methods used and results obtained. The validity and certainty of the results were noted (whether appropriate statistical comparisons were used and, if so, their effect size and significance). The senior author identified and grouped common themes, divided larger themes into subthemes, tabulated a combined summary of the paper, and synthesized a common rubric for each theme. Consolidated reviewers' comments can be found in *Table* *S1* (supporting information).

## Results

**Figure 1:**
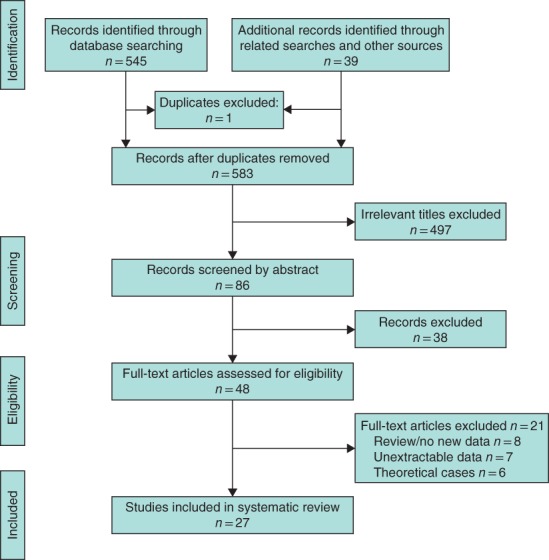
PRISMA diagram for the study

A total of 584 articles were identified from the literature search, of which 48 were retrieved for evaluation. Papers were excluded on the basis of being duplicates (1) and being irrelevant based on the title (497) and abstract (38) (*Fig*. 1). Twenty‐seven studies^16^, ^17^, ^18^, ^19^, ^20^, ^21^, ^22^, ^23^, ^24^, ^32^, ^33^, ^34^, ^35^, ^36^, ^37^, ^38^, ^39^, ^40^, ^41^, ^42^, ^43^, ^44^, ^45^, ^46^, ^47^, ^48^, ^49^ comprising 20 898 patients met the inclusion criteria and were included in the narrative synthesis (*Appendix* *S1*, supporting information).

### Baseline characteristics and study design

Study demographics are shown in *Table* 1. Fourteen papers^16^, ^17^, ^18^, ^19^, ^32^, ^33^, ^34^, ^36^, ^37^, ^40^, ^42^, ^43^, ^46^, ^49^, comprising 11 611 patients, made estimations of outcomes before surgery, eight^20^, ^21^, ^22^, ^35^, ^38^, ^39^, ^41^, ^45^ (6299 patients) made estimations after surgery, and five^23^, ^24^, ^44^, ^47^, ^48^ (2988 patients) did both. Four studies^19^, ^33^, ^36^, ^48^ blinded assessors to the scoring systems that were used as a comparator. Seventeen papers^18^, ^19^, ^22^, ^23^, ^24^, ^32^, ^33^, ^34^, ^35^, ^36^, ^37^, ^38^, ^39^, ^45^, ^47^, ^48^, ^49^ had a NO score of 6 or above. The generic risk prediction tools used in the included studies are detailed in *Appendix* *S2* (supporting information). Twelve studies^17^, ^19^, ^20^, ^22^, ^24^, ^32^, ^36^, ^37^, ^45^, ^47^, ^48^, ^49^ provided AUC values, two^36^, ^41^ provided O : E data, and one^46^ *R* ^2^ data (*Table* 2).

**Table 1:** Demographic data and Newcastle–Ottawa scores of included studies

Reference	No. of patients	Geographical location	No. of centres	No. of surgeons	Consecutive patients	Surgical specialty*	Timing of risk estimation†	Other scoring system(s) used for comparison	NO score‡
Arvidsson *et al*.^16^	1361	Sweden	Single	0	Yes	1, 5	1	No	4
Bakaeen *et al*.^49^	317	USA	Single	9	Yes	4	1	CICSP	6
Burgos *et al*.^17^	232	Spain	Single	3	Yes	5	1	ASA, Barthel index, Goldman index, Charlson index, POSSUM	5
Cornwell *et al*.^18^	181	USA	Single	8	No	4	1	CICSP	6
Farges *et al*.^24^	946	France	Multiple	26	Yes	2	3	Internally validated prediction model	7
Ghomrawi *et al*.^32^	391	USA	Single	8	Yes	5	1	WOMAC	6
Glasgow *et al*.^33^	1791	USA	Multiple	n.d.	No	1, 6	1	NSQIP	7
Graz *et al*.^34^	197	Switzerland	Single	n.d.	Yes	5	1	No	6
Hartley and Sagar^35^	120	UK	Single	2	Yes	2, 3	2	POSSUM	6
Hobson *et al*.^36^	163	UK	Single	n.d.	Yes	1, 6, 9, 10, 11	1	POSSUM, P‐POSSUM	6
Jain *et al*.^37^	5099	USA	Single	n.d.	Yes	4	1	VA mortality risk estimate	7
Kaafarani *et al*.^38^	1622	USA	Multiple	n.d.	n.d.	1	2	No	8
Karliczek *et al*.^39^	191	Netherlands	Single	32	n.d.	2, 3	2	No	7
Lutz *et al*.^40^	273	USA	Multiple	n.d.	No	5, 7	1	No	5
Markus *et al*.^41^	1077	Germany	Single	≥ 5	Yes	2, 3	2	POSSUM, P‐POSSUM	5
Meijerink *et al*.^42^	53	Netherlands	Single	2	n.d.	5	1	KSCRS	5
Pettigrew and Hill^43^	218	New Zealand	Single	n.d.	Yes	2, 3	1	No	5
Pettigrew *et al*.^44^	113	New Zealand	Single	n.d.	Yes	2, 3	3	No	5
Pons *et al*.^19^	1198	Spain	Multiple	n.d.	Yes	4	1	Internally validated prediction model	6
Promberger *et al*.^20^	2558	Austria	Single	14	No	8	2	No	5
Sagberg *et al*.^21^	299	Norway	Single	13	n.d.	7	2	No	5
Samim *et al*.^45^	349	UK and Netherlands	Multiple	12	No	2	2	No	6
Sammour *et al*.^22^	83	Australia	Single	n.d.	Yes	3	2	Anastomotic leak calculator (online calculator)	6
Smith and McCahill^23^	57	USA	Single	n.d.	n.d.	2, 3	3	Internally validated prediction model	6
Timmermans *et al*.^46^	137	Netherlands	Single	4	No	6	1	Markov analysis tool	3
Woodfield *et al*.^47^	1013	New Zealand	Single	58	Yes	2, 3, 6	3	No	6
Woodfield *et al*.^48^	859	New Zealand	Multiple	n.d.	n.d.	2, 3, 6	3	POSSUM, P‐POSSUM	6

* Surgical specialty: 1, general surgery; 2, upper gastrointestinal/hepatopancreatobiliary; 3, colorectal; 4, cardiothoracic; 5, orthopaedic; 6, vascular; 7, neurosurgery; 8, endocrine; 9, urology; 10, renal; 11, gynaecology.

† Timing of risk estimation: 1, preoperative; 2, postoperative; 3, both preoperative and postoperative.

‡ Maximum Newcastle–Ottawa (NO) score is 9. CICSP, Continuous Improvement in Cardiac Surgery Program; POSSUM, Physiological and Operative Severity Score for the enUmeration of Mortality and morbidity; WOMAC, Western Ontario and McMaster Universities Arthritis Index; n.d., no data; NSQIP, National Surgical Quality Improvement Program (American College of Surgeons); P‐POSSUM, Portsmouth POSSUM; VA, Veterans Affairs; KSCRS, Knee Society Clinical Rating System.

**Table 2:** Study‐specific data

Reference	Risk outcome assessed*	Timing of risk estimation†	Type of risk assessment‡	Timing of risk event§	Absolute value of risk event occurrence (%)	Absolute value of risk event predicted by surgeon (%)	Absolute value of risk event predicted by scoring system (%)	Surgeon ROC, AUC, *R* ^2^ or O : E value	Scoring system	Scoring system ROC, AUC, *R* ^2^, or O : E value
Arvidsson *et al*.^16^	2	1	1	1	31	n.d.	n.d.	n.d.	n.d.	n.d.
Bakaeen *et al*.^49^	1	1	2	1	5·4	8·3	6·6	AUC 0·73	CICSP	AUC 0·75
Burgos *et al*.^17^	1	1	1	1	11·2	n.d.	n.d.	AUC 0·677	ASA	AUC 0·600
									Barthel index	AUC 0·689
									Goldman index	AUC 0·432
									Charlson index	AUC 0·590
									POSSUM	AUC 0·635
	2	1	1	1	10·3	n.d.	n.d.	AUC 0·833	ASA	AUC 0·675
									Barthel index	AUC 0·672
									Goldman index	AUC 0·652
									Charlson index	AUC 0·707
									POSSUM	AUC 0·726
	3	1	1	1	73·3	n.d.	n.d.	AUC 0·70	ASA	AUC 0·624
									Barthel index	AUC 0·737
									Goldman index	AUC 0·567
									Charlson index	AUC 0·634
									POSSUM	AUC 0·646
Cornwell *et al*.^18^	1	1	1	1	6·1	12	7·5	n.d.	CICSP	n.d.
				2	11	n.d.	n.d.	n.d.	CICSP	n.d.
Farges *et al*.^24^	1	1	1	3	20·4	44·9	n.d.	AUC 0·76	n.d.	AUC 0·76
		2	1					AUC 0·76	n.d.	AUC 0·83
	2	1	1	3	49·4	38·8	n.d.	AUC 0·77	n.d.	AUC 0·80
		2	1					AUC 0·78	n.d.	AUC 0·81
	3	1	1	3	8 days	30	n.d.	AUC 0·74	n.d.	AUC 0·80
		2	1					AUC 0·75	n.d.	AUC 0·81
Ghomrawi *et al*.^32^	3	1	4	2	90	n.d.	n.d.	n.d.	WOMAC pain	ROC 0·74
									WOMAC function	ROC 0·67
	3	1	4	2	65	n.d.	n.d.	n.d.	WOMAC pain	ROC 0·51
									WOMAC function	ROC 0·51
Glasgow *et al*.^33^	1	1	2	1	n.d.	n.d.	n.d.	n.d.	NSQIP	n.d.
	2	1	1	1	8·2	7·7 (mean)	9 (mean)	n.d.	NSQIP	n.d.
Graz *et al*.^34^	3	1	3	2	17·7	79	n.d.	n.d.	n.d.	n.d.
Hartley and Sagar^35^	2	2	3	1	15·8	24·4	50	n.d.	POSSUM	n.d.
Hobson *et al*.^36^	1	1	1	1	9·2	Surgeon 11	POSSUM 15·3	AUC 0·903	POSSUM	AUC 0·946
								O : E 0·83		O : E 0·6
						Anaesthetist 9·8	P‐POSSUM 9·2	AUC 0·907	P‐POSSUM	AUC 0·940
								O : E 0·93		O : E 1·0
Jain *et al*.^37^	1	1	1	1	3·3	5·6	4·3	AUC 0·73	CICSP	AUC 0·78
Kaafarani *et al*.^38^	2	2	1	3	6·8	n.d.	n.d.	n.d.	n.d.	n.d.
Karliczek *et al*.^39^	2	2	1	1	13·6	7·8	n.d.	n.d.	n.d.	n.d.
Lutz *et al*.^40^	3	1	3	2	61·4	n.d.	n.d.	n.d.	n.d.	n.d.
Markus *et al*.^41^	2	2	2	1	29·5	32·1	46·4	O : E 0·92	POSSUM	O : E 0·64
Meijerink *et al*.^42^	3	2	1	1	n.d.	n.d.	n.d.	n.d.	n.d.	n.d.
	4	1	1	n.d.	n.d.	n.d.	n.d.	n.d.	KSCRS	n.d.
Pettigrew and Hill^43^	2	1	1	1	17·9	n.d.	n.d.	n.d.	n.d.	n.d.
Pettigrew *et al*.^44^	2	1	1	1	25	n.d.	n.d.	n.d.	n.d.	n.d.
		2	1	1	25	n.d.	n.d.	n.d.	n.d.	n.d.
Pons *et al*.^19^	1	1	3	1	10·5	10·8	18·2	AUC 0·70	n.d.	AUC 0·76
Promberger *et al*.^20^	2	2	3	1	28·3	n.d.	n.d.	n.d.	n.d.	AUC 2617
	3	2	3	2	2·5	n.d.	n.d.	n.d.	n.d.	AUC 544·1
Sagberg *et al*.^21^	3	2	1	1	n.d.	n.d.	23	n.d.	n.d.	n.d.
Samim *et al*.^45^	2	1	2	1	55·9	n.d.	n.d.	AUC 0·64	n.d.	n.d.
Sammour *et al*.^22^	2	2	1	1	9·6	5	9	AUC 0·4	Anastomotic leak online	AUC 0·84
Smith and McCahill^23^	3	1	3	3	19·05 months	15·5 months	n.d.	n.d.	n.d.	n.d.
	3	1	3	3	n.d.	n.d.	n.d.	n.d.	n.d.	n.d.
		2	2	3	n.d.	n.d.	n.d.	n.d.	n.d.	n.d.
Timmermans *et al*.^46^	1	2	1	1	6·1	7·3	6·1	*R* ^2^ 0·52–0·91	n.d.	R2 0·98
Woodfield *et al*.^47^	1	1	1	1	3·2	n.d.	n.d.	AUC 0·74	n.d.	n.d.
		2						AUC 0·75	n.d.	n.d.
	2	1		1	14·3	n.d.	n.d.	AUC 0·67	n.d.	n.d.
		2						AUC 0·69	n.d.	n.d.
Woodfield *et al*.^48^	2	1	1 (global VAS)	1	24·1	n.d.	n.d.	AUC 0·778	POSSUM	AUC 0·76
		2						AUC 0·81		
	2	1	1 (multifactorial VAS)	1	18·7	n.d.	n.d.	AUC 0·779	POSSUM	AUC 0·772
		2						AUC 0·89		
	2	1	1 (after feedback)	1	15·8			AUC 0·895	POSSUM	AUC 0·791
		2						AUC 0·918		
	2	1	1 (overall)	1	20·5	n.d.	n.d.	AUC 0·789	POSSUM	AUC 0·754
		2						AUC 0·882		

* Risk outcome assessed: 1, mortality; 2, general morbidity; 3, long‐term outcomes; 4, other.

† Timing of risk estimation: 1, preoperative; 2, postoperative.

‡ Type of risk assessment: 1, continuous scale; 2, quantitative; 3, qualitative; 4, composite scale.

§ Timing of risk event: 1, early postoperative; 2, late postoperative; 3, early and late postoperative. ROC, receiver operating characteristic curve; AUC, area under the curve; O : E, observed : expected; n.d., no data; CICSP, Continuous Improvement in Cardiac Surgery Program; POSSUM, Physiological and Operative Severity Score for the enUmeration of Mortality and morbidity; WOMAC, Western Ontario and McMaster Universities Arthritis Index; NSQIP, National Surgical Quality Improvement Program (American College of Surgeons); P‐POSSUM, Portsmouth POSSUM; KSCRS, Knee Society Clinical Rating System; VAS, visual analogue scale.

### Outcomes

#### *Mortality*

Ten studies, comprising 10 121 patients (9314 preoperative and 3638 postoperative risk estimates), assessed surgeons' predictions of mortality in patients undergoing cardiac^18^, ^19^, ^37^, ^49^, general^23^, ^33^, ^36^, orthopaedic^17^, vascular^46^ and hepatic^24^ surgery. Absolute estimates of mortality were predicted for nine studies^17^, ^18^, ^19^, ^33^, ^36^, ^37^, ^46^, ^47^, ^49^, and ranged from 3·3 to 20·4 per cent (*Table* 2). All studies assessed 30‐day mortality, except one^16^ that assessed 90‐day mortality.

In all but one study^24^, surgeons overestimated the mortality risk. In six of seven studies assessing mortality estimate, surgeons (range 0·68–0·91) were outperformed by risk prediction tools (range 0·64–0·98). The most accurate assessment of mortality risk was in a series of 163 patients undergoing emergency general surgical operations^36^. Both surgeons and anaesthetists assessed risk, with anaesthetists (O : E ratio 0·93; AUC 0·907) performing marginally better than surgeons (O : E ratio 0·83; AUC = 0·903). In cardiac surgery, surgeons rarely classified individuals as low risk, even when they were^19^, ^37^, ^49^. Four papers provided mortality assessments using mortality estimate risk scoring tools (POSSUM 2^17^, ^36^; P‐POSSUM 1^36^; CICSP 2^18^, ^49^). These scoring tools provided a lower, and more accurate, absolute figure for mortality estimates, with a greater AUC value (when given) in all studies.

#### *General morbidity*

Sixteen studies, comprising 12 832 patients (6882 preoperative and 6024 postoperative risk estimates) undergoing general^16^, ^22^, ^24^, ^33^, ^35^, ^38^, ^39^, ^41^, ^43^, ^44^, ^45^, ^47^, ^48^, orthopaedic^16^, ^17^, vascular^33^, ^47^, ^48^, endocrine^20^ and neurosurgical^21^ operations, assessed surgeons' predictions of general postoperative morbidity (*Table* 2). Absolute estimates of morbidity were predicted in seven studies^24^, ^33^, ^34^, ^35^, ^39^, ^41^, ^42^ and ranged from 5 to 38·8 per cent.

Surgeons overestimated risk in three studies^34^, ^35^, ^41^ where data were provided, and underestimated risk in four studies^22^, ^24^, ^33^, ^39^. One study^41^ demonstrated that surgeons overpredicted complications in elective cases and underpredicted complications in emergency cases. Surgeons' accuracy in estimating morbidity varied considerably (AUC 0·4–0·92). The accuracy of prediction tools showed less variability (AUC 0·65–0·84). Surgeons' predictive accuracy was better than prediction tools in three^17^, ^41^, ^48^ of five^17^, ^22^, ^24^, ^41^, ^48^ comparative studies. Four papers provided morbidity estimates using POSSUM^17^, ^35^, ^41^, ^48^ and P‐POSSUM^48^. Surgeons predicted morbidity better than POSSUM, but were comparable with P‐POSSUM. P‐POSSUM was found to be a better predictor than POSSUM by the authors of one study^48^.

#### *Operation‐specific morbidity*

Three studies^20^, ^22^, ^39^ comprising 2832 patients (all risk assessments made after surgery) evaluated operation‐specific morbidity prediction (*Table* 2). Two^22^, ^39^ (274 patients) assessed surgeons' estimate of developing an anastomotic leak after primary anastomosis. Both showed surgeons' estimated leak rate was approximately half the actual leak rate, with a predictive power no better than that from chance alone. One study^22^ found an online prediction tool for anastomotic leak (AUC 0·84, 95 per cent c.i. 0·67 to 1·00) to be superior to surgeons at estimating leak rates (AUC 0·4). Another study^20^ investigated surgeons' ability to predict accurately the risk of postoperative hypocalcaemia (POH) and permanent hypoparathyroidism following thyroid surgery in 2558 patients. Limited data were available, but the more common hypocalcaemia (occurring 28·3 per cent of the time) was better predicted than the less frequent hypoparathyroidism (occurring 2·5 per cent of the time).

#### *Long‐term outcomes*

Nine studies^17^, ^21^, ^23^, ^24^, ^32^, ^34^, ^38^, ^40^, ^42^ (4070 patients; 2096 preoperative and 2939 postoperative risk estimations) reported surgeons' accuracy in predicting longer‐term outcomes, involving patients undergoing orthopaedic^17^, ^32^, ^34^, ^40^, ^42^, general^23^, ^24^, ^38^ and neurosurgical^21^, ^40^ operations. Outcome measures were heterogeneous and included overall function^21^, pain improvement^23^, global outcome impression^34^, ^40^, hernia recurrence rate^38^, length of hospital stay (LOS)^24^ and long‐term survival^17^.

AUC values were poorly reported, but where available ranged from 0·51 to 0·75. A number of studies^17^, ^21^, ^34^, ^40^, ^42^ found that surgeons significantly and consistently overestimated functional, analgesic and overall satisfaction outcomes after spinal, orthopaedic and neurosurgical operations. The only outcomes that were predicted accurately were ambulation at 90 days after emergency hip fracture surgery^17^ and LOS^24^.

### Comparative analysis

#### *Preoperative* versus *postoperative risk assessment/stratification*

Five studies^23^, ^24^, ^44^, ^47^, ^48^, comprising 2988 patients (2988 preoperative and 2931 postoperative risk estimates) undergoing gastrointestinal or vascular surgery, assessed outcomes prediction immediately before and after surgery using the same assessment tools. Outcomes assessed were mortality^23^, ^24^, ^47^, morbidity^24^, ^44^, ^47^, ^48^, LOS^24^ and symptom improvement^23^.

Of the five studies presenting AUC data, four^23^, ^44^, ^47^, ^48^ found that risk perception was better after than before surgery, although some of the improvements were small. One^24^ found no difference in prediction accuracy before and after surgery. One study^47^ demonstrated that patients with a significantly increased risk assessment after surgery (compared with before surgery) had higher mortality (6·3 *versus* 2·4 per cent respectively; *P* = 0·006), major complication (20·1 *versus* 11·0 per cent; *P* = 0·001) and all complications (48·3 *versus* 34·3 per cent; *P* = 0·001) rates.

#### *Surgeon experience: consultant* versus *junior*

Four papers^21^, ^39^, ^41^, ^48^ (2426 patients; 859 preoperative and 2426 postoperative risk assessments) assessed the difference in predictive accuracy between senior surgeons (consultants or attending surgeons) and surgeons in training. Outcomes assessed were morbidity^39^, ^41^, ^48^ and functional status^21^. Three papers^21^, ^39^, ^48^ (gastrointestinal surgery and neurosurgery) found a trend towards better predictions by surgeons in training, whereas one^41^ (elective and emergency major hepatobiliary and gastrointestinal surgery) showed that senior surgeons were better than trainees in predicting outcomes.

## Discussion

This systematic review and narrative synthesis examined the accuracy of surgeons' estimates in predicting outcomes. Surgeons' predictions of mortality in both general and cardiac surgery were good, with most of the AUCs presented in papers being greater than 0·7. Where data were presented, surgeons consistently overestimated mortality risk. Only one paper^36^ assessed anaesthetic risk, and found that anaesthetists predicted mortality following emergency general surgery more accurately than surgeons. In cardiac surgery, surgeons rarely classified individuals as low risk even when they were^19^, ^37^, ^49^. Prediction tools (POSSUM, P‐POSSUM and CICSP) consistently predicted mortality rate more accurately than surgeons, with lower absolute values. P‐POSSUM performed exceptionally well in a single study^36^ of emergency general surgery. Mortality overestimation was a consistent finding in a recent study^50^ in which residents were given real‐life clinical vignettes and asked to estimate risks. It is been suggested that the pessimism in predictions may allow patients to exceed surgeons' expectations (when pessimistic predictions are proven wrong), which is psychologically preferable to patients failing to meet a pre‐established expectation^37^. These findings differ to physicians' estimates of mortality in the ICU. Radtke and colleagues^51^ found that ICU physician estimates were as good as risk assessment tools, and either accurately or slightly underestimated mortality risk.

For general morbidity, surgeons were relatively good at predicting outcomes (AUC generally above 0·6 where data were given). Data on absolute risk rates were not given routinely, and when presented there was no consistent overprediction or underprediction of risk. One study^41^ suggested that surgeons overpredicted complications in elective cases and underpredicted risk in emergency cases. Pre‐existing scoring systems were better than surgeons' predictions in some studies^18^, ^22^, but worse in others^33^, ^35^, ^41^. One study^48^ demonstrated that surgeons' accuracy in predicting complications improved with feedback from previous predictions. General morbidity occurs shortly after surgery and is often audited and scrutinized by the operating surgeon; this provides a constant feedback for fine‐tuning individual surgeons' risk estimation.

Three studies^20^, ^22^, ^39^ investigated surgeons' ability to predict specific surgical complications accurately. Two studies^22^, ^39^ showed that surgeons' predictions of anastomotic leak were exceptionally poor, predicting markedly fewer leaks than occurred, in contrast to a risk prediction tool, which performed well. Although there are several caveats to anastomotic leak predictions, foremost that it is exceptionally unusual to create an anastomosis with an expectation of a leak, the risk assessment tool can be used with good accuracy. The large study by Promberger and co‐workers^20^ showed that a more common complication was better predicted than a less frequent one, perhaps due to better pattern recognition by the surgeons.

Predictions of long‐term outcomes following surgery are variable, in part due to marked heterogeneity, but clearly demonstrate poor predictive power of surgeons. This summary is based predominantly on spinal, orthopaedic and neurosurgical surgery, in which outcomes are recognized as being variable. Although this does limit generalizability, it may also be that surgeons do not routinely follow up patients for a long time (beyond 1 year), and therefore estimates of long‐term outcomes are based on fewer patient encounters than more immediate surgical outcomes. It may also be due to confirmation bias, which is related to the overconfidence hypothesis^52^, when surgeons preferentially remember successful outcomes and forget failures, highlighting the importance of auditing patient outcomes.

This systematic review allowed comparisons between preoperative and postoperative risk predictions, and between senior surgeons and surgeons in training. However, only patients who had a surgical intervention were included, and so this review does not examine the risk assessment of patients managed without surgery, which comprises a large volume of the surgical workload.

A significant weakness of this review was the marked heterogeneity between the included studies, with significant differences in risk assessment methods, statistical analysis, assessment of outcome and data presentation, which precluded meta‐analysis. Additionally, given the limited volume of data, it was impossible to perform separate analyses of individual surgical specialties, despite the risk that postoperative outcomes may be perceived significantly differently between various specialties depending on baseline event rate. Furthermore, the information available to the operating surgeon during risk evaluation was not always apparent and estimates may, therefore, have been prejudiced by the use of scoring schemes (such as P‐POSSUM). Certain studies^24^, ^32^ used subjective outcome measures susceptible to bias. Risk predictions made before and after surgery were grouped together. Finally, a number of studies^16^, ^18^, ^21^, ^23^, ^33^, ^34^, ^35^, ^38^, ^40^, ^41^, ^42^, ^43^, ^44^ did not provide AUC data (or equivalent). It was therefore impossible to make meaningful statistical comparisons between studies, which might have been possible with a more focused review including only studies with AUC data.

This systematic review has several implications for surgical practice. Surgeons need to be aware of the global limitations of surgeons' judgement. The consistent finding of an increased prediction of mortality suggests surgeons tend towards more pessimistic predictions, which will invariably influence surgical decision‐making and patient consent. Recall bias (caused by inconsistencies of recalled events), confirmatory bias (the tendency to interpret new evidence as confirmation of one's existing theories), anchoring bias (preference for reliance on information identified first during information‐gathering), overconfidence bias (when a person's subjective confidence in their judgement is consistently greater than the objective accuracy of those judgements), self‐serving bias (the tendency to attribute positive events to personal ability, whilst attributing negative events to external factors), as well as numerous others^14^, ^15^, ^52^, will hamper the surgeon's ability to predict outcomes accurately. Existing risk scoring tools, especially P‐POSSUM and CICSP, appear to be of significant value and outperform surgeons in their estimation of mortality. However, they invariably cannot capture all variables affecting outcome, and should therefore be used as an adjunct to risk estimation. Recently, a machine‐learning algorithm has been developed to predict postoperative outcomes^53^, with AUCs ranging from 0·82 to 0·94 (99 per cent c.i. 0·81 to 0·94) for morbidity and 0·77 to 0·83 (0·76 to 0·85) for mortality. This tool has the potential of using future data to refine its algorithm automatically and improve its predictive power.

Risk evaluation is a crucial step in the surgeon and patient deciding on whether to have surgery. Detailed interviews have demonstrated that risk evaluation often occurs before a patient is seen for the first time, and has a profound influence on how likely surgery is to be offered and accepted^54^. Randomized data assessing surgeons' responses to various clinical vignettes showed that access to data from a well validated risk calculator reduced the variability of risk estimation and led to more accurate risk prediction^55^. This is crucial as a composite estimate of risk/benefit is a key determinant of a surgeon deciding whether to offer an operation^56^, ^57^. Although this study did not include papers in which patients did not undergo an operative intervention, the implication of these results is that risk prediction tools could be of value in reducing heterogeneity between surgeons' willingness to offer patients surgery.

When making decisions, there is a clear difference between intuitive, unconscious, automatic thought and deliberate, conscious, analytical thought^58^, sometimes referred to as system 1 (rapid intuitive thinking that relies on personal experience, bias and heuristics) and system 2 (time‐consuming deliberate thought requiring focus and dedication) thinking^59^. These systems can be viewed as two ends of a continuum, whereby an expert can move effortlessly from one to the other as the situation requires, described as fluidity. It is likely that unconscious intuition was evaluated predominantly in the included studies, and, where able, compared with a tool that would complement the analytical decision‐making aspect. Senior physicians are recognized as using their intuition far more than a novice, in part to avoid overloading their conscious working memory and reduce the risk of burnout associated with excessive system 2 thinking^60^, ^61^. This review highlights the potential value to be gained by using surgical intuition alongside predictive tools, which would complement deliberate and conscious system 2 thought. This decision‐making can be further enhanced by regular multidisciplinary team case discussions and frequent reviews of surgical morbidity and mortality.

## Disclosure

The authors declare no conflict of interest.

## Supporting information


**Appendix S1**. Supplementary Material
