# Supplementary material for: Systematic review and narrative synthesis of surgeons' perception of postoperative outcomes and risk
Source: BJS Open. 2019 Nov 26;4(1):16–26. doi: 10.1002/bjs5.50233 (PMC6996626; doi:10.1002/bjs5.50233)
Supplement: Supplementary file 1 — Appendix S1. Supplementary Material [file BJS5-4-16-s001.docx]

**BJS5_50233**

**Systematic review and narrative synthesis of surgeons’ perception of postoperative outcomes and risk**

**N. M. Dilaver, B. L. Gwilym, R. Preece, C. P. Twine and D. C. Bosanquet**

# Appendix S1 Narrative synthesis of outcomes in included studies

## Non-cardiac Surgery Mortality Prediction – Elective

Glasgow et al **(**2014) reported on surgeons pre-operative predicted mortality rates in 1,791 elective general surgery patients, compared to an internally validated prediction risk model (devised internally by the authors).^33^ Actual 30-day mortality rates were 0.2%. The surgeons' median predicted mortality rates were 1.5% (Interquartile range [IQR]; 0.8-3.5%), whilst the risk models’ were 1.3% (IQR; 0.4-2.3%). Farges et al (2014) evaluated risk prediction for 946 patients undergoing elective hepatectomy pre- and post-operatively, and compared with an internally created prognostic model.^24^ The surgeons' pre- and post-operative mortality prediction AUC was 0.76 (95% CI: 0.66-0.96), and 0.76 (95% CI: 0.66-0.96) respectively, compared to the prognostic model AUC 0.79 (95% CI: 0.70-1.00), and AUC 0.83 (95% CI: 0.74-1.00). Surgeons more frequently underestimated (rather than overestimated) the risks of surgery. Smith et al (2008) prospectively assessed surgeons’ ability to predict estimated survival time in 57 patients with advanced malignancies.^23^ Surgeons reasonably predicted mortality rates post-operatively.

## Non-cardiac Surgery Mortality Prediction – Emergency +/- Elective

Woodfield et al (2007) assessed surgeons' predictions of mortality in 1,013 patients undergoing elective and emergency colorectal, upper gastrointestinal and vascular surgery preoperatively and immediately post-operatively.^47^ Mortality rates were 3.2%. Surgeon AUC values for mortality pre- and post-operatively were similar (0.74 and 0.75 respectively). However, cases in which the risk score increased significantly (defined as an increase greater than 5 mm in VAS score) post-operatively had a greater mortality rate (6.3% versus 2.4%; P = 0.006).^47^ Hobson et al (2007) collected prospective data on 163 patients undergoing emergency general surgery over a four-month period.^35^ Preoperatively the surgeon and anaesthetist estimated the patients’ 30-day mortality risk, and compared this with POSSUM and P-POSSUM scoring systems.^36^ Actual mortality was 9.2%. Both surgeons (predicted 11% mortality; O:E = 0.83; AUC = 0.903) and anaesthetists (predicted 9.8% mortality; O:E = 0.93; AUC = 0.907) over predicted risk, but with AUCs indicative of high performance. P-POSSUM (9.2%; O:E = 1; AUC = 0.940) and POSSUM (15.3%; O:E = 0.6; AUC = 0.946) were very accurate predictors of outcome.^36^ Burgos et al (2007) predicted 90-day mortality rates in 232 patients undergoing hip fracture surgery pre-operatively, and compared with several risk models (ASA, Barthel index, Goldman index, POSSUM, Charlson index and the VAS for Risk scale).^17^ 90-day mortality was 11.2%. Surgeons estimates of mortality had an AUC of 0.68.^17^ Timmermans et al (1996) evaluated surgeons' estimates of operative mortality rates for repair of abdominal aortic aneurysms (AAA) in 137 patients.^46^ The actual mortality rate was 6.1%, with surgeons slightly overestimating mortality at 7.3%.^46^

## Cardiac Surgery Mortality Prediction

Jain et al (2014) asked surgeons to predict 30-day mortality in 5,099 patients undergoing coronary artery bypass grafting (CABG) or valve surgery,^37^ and compared to the Continuous Improvement in Cardiac Surgery Program (CICSP) mortality estimate.^5^ Absolute 30-day mortality was 3.3%. Preoperative 30-day mortality was predicted by surgeons to be 5.6% with an AUC of 0.73 (95% CI; 0.69-0.77), and by CICSP to be 4.3% with an AUC of 0.78 (95% CI; 0.75–0.82).^37^ Bakaeen et al (2010) employed similar methodology to Jain et al ^37^ but limited data to aortic valve replacement (AVR) surgery in 317 patients.^49^ The absolute mortality rate was 5.4%. The surgeons’ mean estimates of mortality was 8.3% (± 8.3%) with an AUC of 0.73, compared to CICSP model estimate of 6.6% (± 8.3%) with an AUC of 0.75.^49^ Another study employing similar methodology examined 181 patients.^18^ 30-day mortality was 6.1%. Surgeons’ predicted mortality was 12%, whilst the CICSP model predicted mortality at 7.5%.^18^ Pons et al (1999) evaluated the predictive accuracy of surgeons’ preoperative mortality risk assessment of 1,198 patients undergoing cardiac surgery compared with an internally created predictive risk model.^19^ The predicted mortality risk was divided into 'low', 'fair', 'high', 'very high' or 'extremely high' risk. The 30-day mortality rate was 10.5%.^19^ The surgeons’ AUC was 0.70, compared to the risk tools’ AUC of 0.76.^19^ Surgeons rarely classified a patient as low risk which led to central clustering of data.^19^

## Short-term outcomes – general morbidity

Farges et al (see above) assessed surgeon estimates of 90-day morbidity pre- and post- hepatectomy and compared them to prognostic models.^24^ Pre-operative predictions by surgeons for morbidity (AUC = 0.77) and severe morbidity (AUC = 0.76) were good, with minimal change post-operatively. The pre-operative prognostic model was more accurate than surgeons (morbidity AUC = 0.80; severe morbidity AUC = 0.81), with minimal improvements noted in the post-operative model.^24^ Samim et al (2018) asked surgeons to pre-operatively predict the risk of developing a major complication for 349 patients undergoing hepato-pancreato-biliary surgery, compared to nine other risk prediction models.^45^ Surgeons' risk assessment was good in liver surgery (AUC = 0.71) and outperformed all but one risk prediction model (Bernstein, AUC = 0.73), however surgeons performed poorly in estimating risk for pancreatic surgery (AUC = 0.56) as did the prediction models (AUC = 0.51-0.57).^45^

Woodfield et al (see above) assessed surgeons risk predictions of severe adverse events pre- and post-operatively using a VAS.^47^ AUC values for major complication pre- and post-operatively were 0.67, 0.69 respectively. The greater the VAS value, the more severe the complication, and when VAS scores were increased greater than 5 mm (comparing pre- and post-operatively) patients had a greater major (20.1% vs 11.0%; P = 0.001), and overall (48.3% vs 34.3%; P = 0.001) complication rate.^47^ In a later study by the same authors, with similar design, Woodfield et al (2017) evaluated surgeons' pre- and post-operative predictions of severe adverse events in 859 patients undergoing colorectal, UGI, and vascular procedures, and compared them with POSSUM and P-POSSUM.^48^ After approximately half the patients were enrolled, surgeons received feedback on their predictive accuracy from the first part of the study. Surgeons' overall predictive accuracy was good, especially post-operatively (pre-operative AUC = 0.778, post-operative AUC = 0.810), although they overestimated risk. They were more accurate following feedback (pre-operative AUC = 0.895; post-operative AUC = 0.918). P-POSSUM was a better predictor than POSSUM, and was comparable to the surgeons’ pre- and post-operative predictions.

Markus et al (2009) studied 1,077 consecutive patients undergoing elective and emergency major hepatobiliary or gastrointestinal surgery.^41^ Surgeons made an immediate post-operative prediction of risk, and compared to POSSUM.^41^ Overall morbidity rates were 29.5%. Surgeons' prediction rates were 32.1% (O:E = 0.92) and the POSSUM score rates were 46.4% (O:E = 0.64).^41^ In general surgeons over predicted complications in elective cases (O:E = 0.77), and under predicted risk in emergency cases (O:E = 1.26).^41^

Burgos et al (see above) assessed surgeons’ predictions of serious complications for 232 patients undergoing hip fracture surgery. Surgeons VAS assessment of risk for incidence of serious complications was good (AUC = 0.833), outperforming other risk prediction tools.^17^ Hartley et al (1994) assessed surgeons’ post-operative predictions of complications for 120 patients undergoing gastrointestinal surgery at a single centre. Surgeons’ post-operative predictions were better compared to POSSUM (no AUC data).^35^

Arvidsson et al (1996) assessed 1,361 patients undergoing general surgical, vascular, urology or orthopaedic operations.^16^ Surgeons made a pre-operative prediction of the risk of an ‘adverse event’ using a VAS. An adverse event occurred 31% of the time. ROC curves were given without AUCs but these show surgeon estimates to be better than ASA, age or procedure magnitude. Glasgow, 2014 et al (see above) also assessed severe morbidity, but without easily comparable outcome data.^33^ The overall morbidity rate was 8.2%. The surgeons’ mean estimate was 7.7%, and the model’s was 9%.^33^ A similar small study with poor reporting of data assessed pre- and post-operative risk scores for 113 patients.^44^ ROCs show post-operative assessment being superior to pre-operative assessment, with an estimated AUC of 0.6-0.7.^44^ The same authors showed similar results in surgeons' predictive accuracy in 218 patients undergoing major gastrointestinal surgery.^43^ Surgeons' assessment had an overall predictive value of 75%, comparable to risk models used for comparison (range: 74-78%).^43^

## Operation specific complication – anastomotic leak

Sammour et al (2016) enrolled 83 consecutive patients in a single centre study of patients undergoing elective or emergency cancer resection with primary anastomosis, excluding those who had formation of a protective stoma.^22^ Post-operatively, the surgeon estimated the risk of developing an anastomotic leak, and this was compared to an online calculator which also provided a risk estimate. The absolute occurrence of anastomotic leak was 9.6%. The surgeons’ predicted a leak rate was 5.0% (AUC = 0.40; 95% CI; 0.21–0.60) whilst the risk calculators prediction was 9% (AUC = 0.84; 95% CI; 0.67–1.00).^22^ In a similar trial, Karliczek et al (2009) included 191 patients undergoing colorectal resection with primary anastomosis.^39^ The absolute occurrence of anastomotic leak was 13.6%. The surgeons' predicted rate was 7.8%. ROCs show the predictive power of surgeons to be no better than chance alone.^39^

## Operation specific complication – other

Promberger et al (2014) investigated the surgeons’ ability to accurately predict the risk of post-operative hypocalcaemia (POH) and permanent hypoparathyroidism (PEH) following thyroid surgery in 2,558 patients, in comparison to a multivariate analysis.^20^ The absolute occurrence of POH and PEH was 28.3% and 2.5% respectively. Data are difficult to extract but demonstrate the surgeons' risk assessment was an independent predictor of risk when included in the model. The more common POH was better predicted than the less frequent occurrence of PEH.^20^

## Long-term outcomes

There were nine studies which reported on longer-term outcomes and the accuracy of the surgeon to predict such outcomes, and how the surgeons’ predictions compare to predictions by scoring tools or risk models.^17,21,23,24,32,34,38,40,42^

Burgos et al (see above) assessed surgeons' predictions of ambulation at 90-days in 232 hip fracture patients.^17^ Ambulation at 90-days was achieved in 73.3% of patients, and surgeons predicted this accurately (AUC = 0.700), performing better than other prediction models except the Barthel index (AUC = 0.737). Ghomrawi et al (2017) assessed surgeons’ predictions of a clinically important improvement in a validated patient-reported outcomes score following total hip arthroplasty (THA; 200 patients) and total knee arthroplasty (TKA; 191 patients).^32^ For THA surgeons’ accuracy was low to moderate at predicting patients who would report improved function (AUC = 0.67, 95% CI; 0.53–0.82) and pain (AUC = 0.74, 95% CI; 0.63–0.85). However, for TKA, surgeons’ predictions were no better than chance (improved function AUC = 0.51, 95% CI; 0.42–0.61, improved pain AUC = 0.51, 95% CI; 0.40–0.61). Meijerink et al (2009) also assessed prediction of outcome after 53 TKAs. Scant data are provided; however, it is clearly shown that at 1-year post-operatively, surgeons' assessment failed to predict subjective or objective patient outcomes.^42^

Sagberg et al (2016) assessed the accuracy of operating neurosurgeons’ predictions about patients’ functional levels 30-days after intracranial tumour surgery on 299 patients, using the Karnofsky Performance Scale (KPS).^21^ The expected KPS score was compared with the observed KPS score at 30-days. Very limited data are presented, but surgeons overestimated their patients’ future functional level in 62% of cases (accurately estimated =15%; underestimated = 23%).^21^

Lutz et al (1999) investigated the improvement in QOL which surgeons' predicted in 273 patients undergoing sciatica surgery.^40^ Satisfaction in changes in symptoms and functional status were measured 1-year post-surgery. The limited data clearly show surgeons overestimate functional recovery: when surgeons' predicted a “great deal of improvement”, 39% of patients were not satisfied with the outcomes and 25% said their symptoms had not improved.^40^ Graz et al (2005) also looked at prediction of outcomes following surgery for sciatica in 197 patients.^34^ Surgeons again overestimated improvements significantly, and predicted a “great improvement” in 79% of patients and “moderate improvement” in 20%; however, 39% of patients had no “minimal clinically important difference” post-surgery.^34^

Kaafarani et al (2005) assessed operating surgeons' satisfaction in 1,622 open or laparoscopic inguinal hernia repairs.^38^ Data presented are limited, but complication rates and recurrence rates at 2 years were not predicted by the surgeons’ assessment.

Farges et al (see above), also assessed surgeons’ predictive power on length of hospital stay (LOS).^24^ Surgeons predicted LOS well both pre- (AUC = 0.74, 95% CI: 0.64-0.93) and post-operatively (AUC = 0.75, 95% CI: 0.64-0.93), but were out-performed by the prognostic models.^24^

Smith et al (see above) prospectively assessed surgeons’ ability to predict estimated survival time and likelihood of symptom relief in 57 patients pre- and post-surgery for advanced malignancy. Preoperatively, surgeons tended to underestimate their patients’ symptom relief, whilst postoperative estimates agreed with patients’ self-assessments.^23^

# Appendix S2 Generic risk prediction tools used by the included studies

1. Continuous Improvement in Cardiac Surgery Programme (CICSP)

*Grover FL, Johnson RR, Shroyer AL, et al. The Veterans Affairs Continuous Improvement in Cardiac Surgery Study. Ann Thorac Surg 1994;58:1845*

The CICSP risk model generates correlation coefficients based on a number of preoperative risk factors including age, prior myocardial infarction, peripheral vascular disease, chronic obstructive pulmonary disease, New York Heart Association functional class, American Society of Anesthesiologists class, diabetes, functional status, smoking, active endocarditis, cardiomegaly, priority of surgery, preoperative mechanical circulatory support, prior heart surgery, plasma creatinine level, resting ST depression on electrocardiography, and current diuretic and digoxin use.

1. American Society of Anesthesiologists Grading System (ASA)

*U Wolters, T Wolf, H Stützer, T Schröder; ASA classification and perioperative variables as predictors of postoperative outcome.,*BJA 1996; *77(2): 217–222*

A five-tier classification for assessing preoperative fitness for surgery:

I: Healthy patient

II: Mild systemic disease

III: Severe systemic disease

IV: Severe systemic disease that pose a constant threat to life

V: Moribund patient not expected to survive without an operation

1. Physiological and Operative Severity Score for the enumeration of Mortality and morbidity (POSSUM)

*Copeland GP, Jones D & Walters M. POSSUM: a scoring system for surgical audit. Br J Surg (1991) 78:356-360*

Scoring system based on objective physiological (age, cardiorespiratory symptoms, vital signs, blood counts/electrolytes and electrocardiogram findings) and operative (severity, blood loss, number of procedures, soiling, malignancy and urgency) criteria that is designed to evaluate morbidity and mortality in surgical patients.

1. Portsmouth Physiological and Operative Severity Score for the enUmeration of Mortality and morbidity (P-POSSUM)

*D.Prytherch et al. POSSUM and Portsmouth POSSUM for predicting mortality. Physiological and Operative Severity Score for the enUmeration of Mortality and morbidity. Br J Surg (1998) 85:1217-1220*

A linear analysis modification of the original POSSUM equation in an attempt to reduce to over-prediction of mortality rates obtained when using the POSSUM formula.

1. American College of Surgeons National Surgical Quality Improvement Program (ACS-NSQIP)

*Bilimoria KY, Liu Y, Paruch JL, Zhou L, Kmiecik TE, Ko CY, Cohen ME. Development and Evaluation of the Universal ACS NSQIP Surgical Risk Calculator: A Decision Aide and Informed Consent Tool for Patients and Surgeons. J Am Coll Surg. 2013 Nov; 217(5): 833–842.e3*

A nationally validated, risk-adjusted, outcomes-based program that predicts the risk of post-operative complications. Factors accounted for include operation type, age, BMI, functional status, ASA, urgency, medical co-morbidities, sepsis status, ventilator dependence and malignancy status.

1. Western Ontario and McMaster Universities Osteoarthritis Index (WOMAC)

*Bellamy N, Buchanan WW, Goldsmith CH, Campbell J, Stitt LW. Validation study of WOMAC: a health status instrument for measuring clinically important patient relevant outcomes to antirheumatic drug therapy in patients with osteoarthritis of the hip or knee. J Rheumatol. 1988 Dec;15(12):1833-40*

A validated 24-item self-administered questionnaire used to evaluate three domains of osteoarthritis; joint pain, stiffness, and physical functioning.

1. Barthel Index

*Mahoney F & Barthel D. Functional evaluation: the Barthel Index. Md Med J. 1965 14: 61–65*

An ordinal scale assessing individuals’ ability to complete ten activities of daily living (eg. walking, dressing).

1. Goldman Index (Cardiac Risk Index)

*Goldman L, Caldera DL, Nussbaum SR et al. Multifactorial Index of Cardiac Risk in Noncardiac Surgical Procedures. NEJM 1977 297 (16): 845–850*

A validated tool used to estimate a patient’s risk of perioperative cardiac complications based on their age, operation type, co-morbidities and bloods results.

1. Charlson Comorbidity Index

*Charlson M, Pompei P, Ales K, MacKenzie RC. A new method of classifying prognostic comorbidity in longitudinal studies: Development and validation. Journal of Chronic Diseases 1987 40 (5): 373 - 83*

A scoring tool that predicts one-year mortality for patients with various comorbidities. The index assesses a total of 22 conditions including cardiac disease, malignancy and human immunodeficiency virus.

1. Knee Society Clinical Rating Scale (KSCRS)

*Insall JN, Dorr LD, Scott RD, Scott WN. Rationale of the*Knee Society clinical rating system*. Clin Orthop Relat Res. 1989 Nov;(248):13-4*

A validated knee scoring system that provides a dual rating system based on a knee score and general functional score (walking and stair ability) designed to remove the problem of declining knee scores associated with patient infirmity.

1. Anastomotic Leak Calculator

*Frasson M et al. Risk Factors for Anastomotic Leak After Colon Resection for Cancer: Multivariate Analysis and Nomogram From a Multicentric, Prospective, National Study With 3193 Patients. Ann Surg 2015: 262: 321-30*

Validated calculator aimed at predicting risk of anastomotic leak in patients undergoing resection of colorectal cancer further than 15cm from the anal verge treated with elective or emergency surgery.

**Table S1** Consolidated reviewers’ comments

| **First Author (Year)** | **Consolidated reviewers’ comments** |
| --- | --- |
| Arvidsson (1996) | • RISK-VAS was the most efficient predictor of early severe events. • ASA is correlated with almost any adverse event, most likely due to the fact that it is a comprehensive descriptor of the patients’ general condition. • Patient related variables such as ASA, class, and age appear to identify severe adverse events more efficiently than mild adverse events. • For all systems, the sensitivity at a given level was always better the more severe the adverse event that was sought. This is not surprising since adverse events of low severity are generally regarded as “noise”, more or less unpredictable, in which the more severe events are embedded. This selectivity may even be regarded as a clinical advantage. • The four classification systems performed equally well in their ability to discriminate between patients who subsequently will or will not have a postoperative adverse event. • The paper concluded that none of the systems are ideal for the counselling of individual patients. All have moderate sensitivity and specificity and too low predictive values. |
| Bakaeen (2010) | • Both surgeons and the CICSP model performed well in risk stratifying aortic valve replacement (AVR) patients, however, when it comes to the absolute quantification of risk, the surgeon tended to overestimate operative mortality in comparison with the risk model. • Surgeons tended to overestimate mortality risk when they anticipated an extensive operation (i.e. performing a mitral valve repair or root enlargement in addition to AVR). • The CICSP model did not capture some disease entities considered relevant in estimating mortality by surgeons. • Certain conditions are not captured or adjusted for by the CICSP model (i.e. bleeding disorders, liver cirrhosis) and hence may be deemed low-risk for AVR by the model, but in reality, these are high risk patients. • Overall, the CICSP model performed better than the surgeons in the study. |
| Burgos (2008) | • RISK-VAS scale was the best predictor of serious post-operative complications during the hospital stay and had sufficient specificity and sensitivity in the prediction of ambulation at 3 months, but not as a predictor of mortality. • All six scoring systems studied for risk assessment or physiological evaluation were proven to be unreliable predictors of serious postoperative complications, functional recovery and 3-month survival. • ASA had no predictive value for any of the response variables. • Goldman index (index to assess cardiovascular risk in non-cardiac surgery) was not found to have prognostic relevance. • POSSUM was relevant for the prediction of serious complications, but not for the evaluation of 90-day mortality or functional recovery. |
| Cornwell (2012) | • CICSP risk assessment to predict operative mortality (30 day and in-hospital mortality) was shown to be accurate in this study. • In high-risk CABG cases the surgeon and CICSP risk estimates often disagreed markedly. • Mean mortality prediction by surgeon was higher than the risk model. • Surgeons estimate of mortality risk was more accurate when duration of mortality risk was extended up to 180 days after surgery, although no data provided. • Mortality nearly doubled at 180-days as 9 additional patients died between 30 and 180-days after surgery. 7 of the 9 patients were deemed high risk by the surgeon but not by the CICSP risk model, and all 9 had additional factors of concern to the surgeon that were not accounted for by the model. Only 2 of those patients died of cardiac causes. |
| Farges (2014) | • Surgeons aren’t accurate with their predictions. • Surgeons’ preoperative anticipation of individual patients’ postoperative outcome had low sensitivity, specificity, and accuracy, which was not improved by surgical experience. • Surgeons underestimated the risk of complications and LOS. • Surgeons anticipation of the difficulty of the procedure was good, however this did not correlate with clinical outcome.  • Models were better than surgeons’ subjective anticipation and combining the model with the surgeon’s subjective opinion did not improve the accuracy of the predictions. • Surgeons should be aware of the limitations of their subjective anticipation and take this into account when counselling patients. |
| Ghomrawi (2017) | • Most patients having total hip arthroplasty (THA) and total knee arthroplasty (TKA) achieved the MCID improvement after surgery. • Surgeons’ were unable to accurately discriminate between patients who benefit and those who do not benefit from scheduled THA. • Surgeons’ expectations were reasonably good in discriminating between patients who did not do well versus those who did well after THA. • Surgeons’ expectation scores did not discriminate between good and poor outcomes in patients undergoing TKA. • Surgeons’ expectations are reasonably predictive of improvement for patients undergoing THA who are 65 years or older, have one or more comorbidities, and who are male, but surgeons’ expectations do not appear to anticipate results as accurately in other THA groups, nor in patients undergoing TKA. |
| Glasgow (2014) | • Across a broad range of general surgical operations, model risk estimates are good compared to risk estimates of experienced surgeons at identifying patients at high risk for morbidity and mortality. • In comparing the predicted (model or surgeon) to observed overall morbidity for each variable category, both the model and surgeon consistently underestimated the overall morbidity. • In regard to functional status, both the model and surgeon predicted a higher associated morbidity than observed. • For the majority of the patient characteristics, the model risk prediction was better at predicting the overall morbidity rate than the surgeons. • For upper gastrointestinal and hepatopancreaticobiliary operations, the statistical model and surgeons overestimated risk of morbidity. • For Colorectal, vascular, cholecystectomy, skin and hernia operations the model and surgeons underestimated risk. |
| Graz (2005) | • Surgeons tended to give overly optimistic predictions that were not correlated with patient outcome.  • More optimistic physician expectation was associated with better improvement of psychosocial dimensions. |
| Hartley (1994) | • The surgeons gut feeling post-operatively is a good predictor of the post-operative course of a patient. • Surgeons tended to be more pessimistic when predicting complications • The surgeons ‘gut feeling’ is generally a better predictor of post-operative complications. |
| Hobson (2007) | • P-POSSUM gave the most accurate prediction of 30-day mortality using linear analysis and POSSUM gave the most accurate prediction using exponential analysis. • Clinical judgement of mortality from both surgeons and anaesthetists were similar to predictions provided by scoring systems for 30-day mortality, however surgeons may underestimate mortality in very high-risk patients. • Anaesthetists were more accurate than surgeons with their predictions. |
| Jain (2014) | • The risk model was better at predicting operative and long-term mortality compared with physician’s subjective risk estimate. • Both physicians and the risk estimation tools modestly overestimated actual operative mortality risk. • Physician’s risk estimate of mortality was higher than statistical risk estimate across all subgroups, except in the highest-risk patients, where physicians underestimated mortality risk. |
| Kaafarani (2005) | • Surgeons were significantly more frustrated and less satisfied with the laparoscopic technique. • The level of a surgeon’s frustration during an inguinal herniorrhaphy was a better predictor of outcomes of the operation than was satisfaction with the procedure. • Frustration was strongly related to the incidence of post-op complications, especially intra-operative. • (Open repair) A procedure in which the surgeon reported being frustrated had double the risk of recurrence within 2 years than a procedure in which the surgeon reported no frustration (sources of frustration was most commonly reported as patient anatomy). • (Lap repair) No association between recurrence and frustration. • Surgeon’s satisfaction with the hernia repair was not predictive of postoperative complications or recurrence in either an open or lap repair. |
| Karliczek (2009) | • Surgeons’ clinical risk assessment had a low predictive value for anastomotic leakage in gastrointestinal surgery. • Clinical judgement of the operating surgeon appeared to have low sensitivity and specificity for all anastomoses. • No reliable intra-operative predictive test for anastomotic leakage by the operating surgeon. |
| Lutz (1999) | • Patients self-reported results, hence biased results. Patients expectations however appear to be important predictors of outcomes. • Surgeons overestimated the benefit of discectomy for patients. |
| Markus (2005) | • Surgeons overpredicted rate of complications in elective cases and underestimated in emergency cases. • Surgeons outperformed POSSUM in predicting morbidity. |
| Meijerink (2008) | • Poor correlation between immediate post-operative surgeon satisfaction and patient satisfaction at 1 year. • Surgeon satisfaction at 1-year remained very similar to immediate post-operative satisfaction. • Prediction of difficulty of procedure by surgeon pre-operatively also had poor correlation with all measured outcomes. |
| Pettigrew (1986) | • Surgeons’ were able to identify those at very high risk but performed worse than their comparators and a simple clinical (medical) assessment overall. • Some 'standardisation' of perceived risk cannot be excluded in this study, the most experienced of the 8 surgeons involved in this study (with the lowest complication rate) had the same trend of recorded perceived risk as the other 7. |
| Pettigrew (1987) | • Post-operative surgeon assessment appears best predictor of morbidity, followed by pre-operative surgeon assessment, both appear superior to medical assessment and albumin levels. |
| Pons (1999) | • Surgeons were good at predicting mortality overall, but some centres outperformed the others. • Surgeons performed best for patients at the two extremes of risk. • The model can improve predictions of surgeons in most assessed centres. |
| Promberger (2014) | • Individual risk assessment scored by the surgeons directly after the operation provided useful predictive information but this was less predictive than a the risk model. • The more common post-operative hypocalcaemia (POH) was more reliably predicted than the less frequent complication of define. • Individual surgeons also differed in their ability to predict the risk of POH and permanent hypoparathyroidism. |
| Sagberg (2016) | • Surgeons' tendency to overestimate functional level overall. • Overestimation was associated with unexpected post-operative complications. • No difference between accuracy of consultants and residents predictions. |
| Samim (2018) | • Surgeons’ assessment showed fair agreement between the observed and predicted probabilities for liver surgery, however agreement was poor for pancreas surgery. • Only one risk prediction model for liver surgery (Bernstein) was superior to clinical assessment but this difference was not significant. • Overall surgeons’ assessment outperformed all but one risk prediction model for liver surgery but ultimately both surgeons & risk models have poor predictive performance for pancreatic surgery. |
| Sammour (2016) | • Calculator provided accurate prediction of anastomotic leak • Surgeons were not accurate at predicting anastomotic leak. |
| Smith (2008) | • Surgeons were more accurate at predicting survival time if considering the patients as not having the planned surgery compared to estimating their survival time with the planned procedure. • Pre-operative prediction of symptomatic improvement did not correlate well with patient self-evaluation, but were accurate at evaluating palliative success post-operatively (correlated with patient self-assessment). |
| Timmermans (1996) | • Average probability estimates are accurate, but not quite as good as the model. • Surgeons tended to underestimate high risk patients' mortality and overestimate low risk patients' mortality. |
| Woodfield (2007) | • Surgeons make meaningful pre-operative prediction of major complications. • Surgeons often increase VAS score post-operatively, this identifies significantly more complications, but the improvement in the discrimination was small. • When included in a multivariate model for predicting post-operative complications, the surgeon’s VAS score functioned as an independent predictive variable and improved the predictive ability, goodness of fit, and discrimination of the model. |
| Woodfield (2017) | • Postoperative predictions comparable to POSSUM and overall better than pre-operative predictions. • Surgeons predictions were best for high risk patients, with a tendency to overestimate risk in the rest of the patients. • Feedback on previous accuracy of predictions improved future predictions of surgeons’ both pre- and post-operatively. |
